# CAR race to cancer immunotherapy: from CAR T, CAR NK to CAR macrophage therapy

**DOI:** 10.1186/s13046-022-02327-z

**Published:** 2022-03-31

**Authors:** Kevin Pan, Hizra Farrukh, Veera Chandra Sekhar Reddy Chittepu, Huihong Xu, Chong-xian Pan, Zheng Zhu

**Affiliations:** 1 Vanderbilt University, 2201 West End Ave, Nashville, TN 37235 USA; 2 Brigham and Women’s Hospital, Harvard Medical School, Boston, MA USA; 3 Boston University, Boston, MA USA; 4 VA Boston Healthcare System, West Roxbury, MA USA; 5 Harvard Medical School, 1400 VFW Parkway Building 3, Room 2B-110, West Roxbury, MA 02132 USA

**Keywords:** Chimeric antigen receptor (CAR), CAR macrophage, CAR T therapy, CAR NK cells, Adoptive cell transfer, Immunotherapy, Tumor microenvironment, Cytokine release syndrome

## Abstract

Adoptive cell therapy with chimeric antigen receptor (CAR) immunotherapy has made tremendous progress with five CAR T therapies approved by the US Food and Drug Administration for hematological malignancies. However, CAR immunotherapy in solid tumors lags significantly behind. Some of the major hurdles for CAR immunotherapy in solid tumors include CAR T cell manufacturing, lack of tumor-specific antigens, inefficient CAR T cell trafficking and infiltration into tumor sites, immunosuppressive tumor microenvironment (TME), therapy-associated toxicity, and antigen escape. CAR Natural Killer (NK) cells have several advantages over CAR T cells as the NK cells can be manufactured from pre-existing cell lines or allogeneic NK cells with unmatched major histocompatibility complex (MHC); can kill cancer cells through both CAR-dependent and CAR-independent pathways; and have less toxicity, especially cytokine-release syndrome and neurotoxicity. At least one clinical trial showed the efficacy and tolerability of CAR NK cell therapy. Macrophages can efficiently infiltrate into tumors, are major immune regulators and abundantly present in TME. The immunosuppressive M2 macrophages are at least as efficient as the proinflammatory M1 macrophages in phagocytosis of target cells; and M2 macrophages can be induced to differentiate to the M1 phenotype. Consequently, there is significant interest in developing CAR macrophages for cancer immunotherapy to overcome some major hurdles associated with CAR T/NK therapy, especially in solid tumors. Nevertheless, both CAR NK and CAR macrophages have their own limitations. This comprehensive review article will discuss the current status and the major hurdles associated with CAR T and CAR NK therapy, followed by the structure and cutting-edge research of developing CAR macrophages as cancer-specific phagocytes, antigen presenters, immunostimulators, and TME modifiers.

**Supplementary Information:**

The online version contains supplementary material available at 10.1186/s13046-022-02327-z.

## Background

**Fig. 1:**
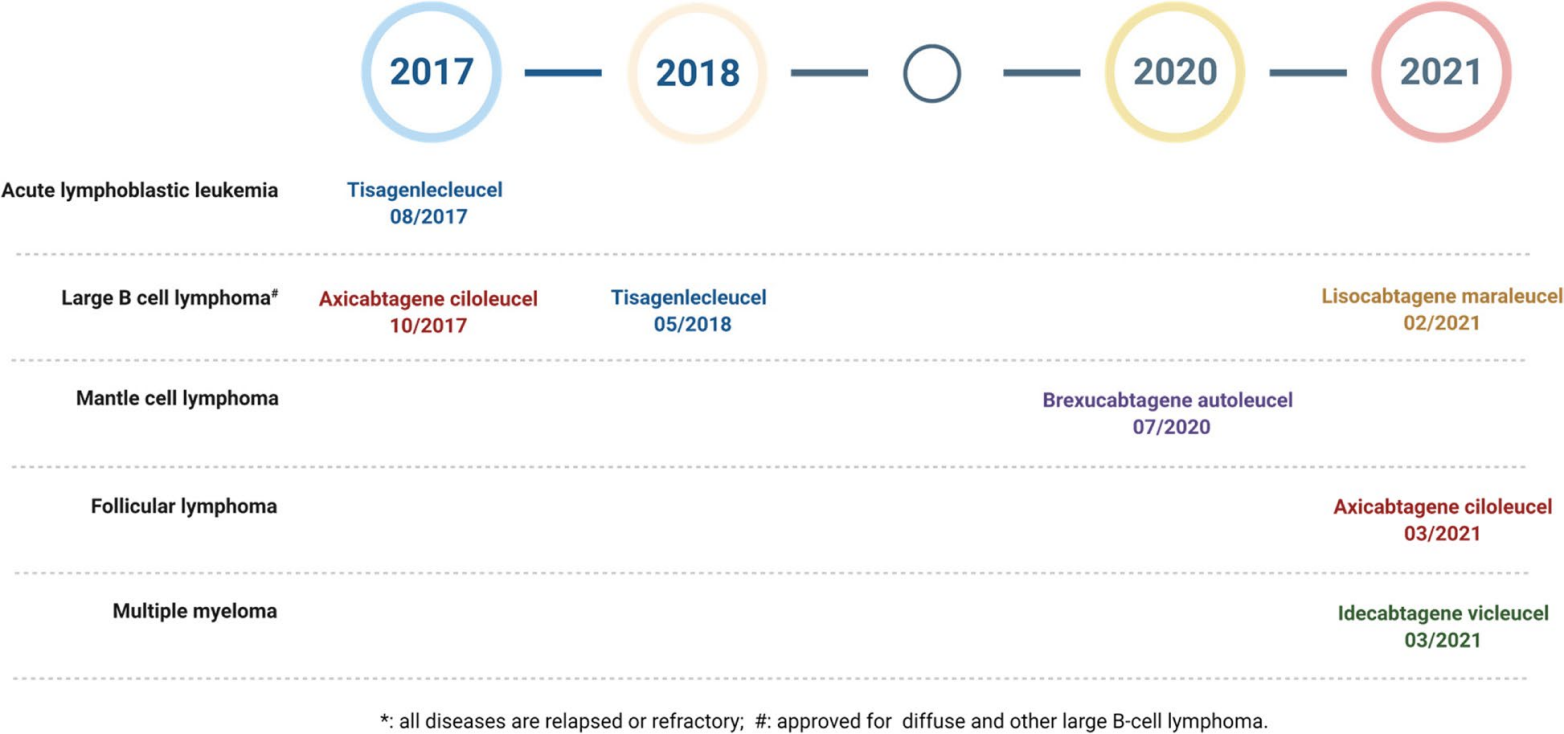
Timeline of CAR T therapy FDA approvals

Immunotherapy has recently revolutionized cancer treatment and constitutes the fourth cornerstone of cancer therapy after surgery, radiation and chemotherapy. This is especially true for metastatic cancers which are usually considered incurable, but, now with immunotherapy, some patients can achieve long-term remission and possibly cure. Currently immunotherapy research explores and harnesses every aspect of the immune system with the most successful stories on immune checkpoint inhibitors (ICIs) and adoptive cell therapy (ACT) with chimeric antigen receptor (CAR) T cells. Other immunotherapy approaches are mainly at preclinical research and clinical trials. For ICIs, one antibody targeting Cytotoxic T Lymphocyte-Associated Protein 4 (CTLA-4), six anti-programmed cell death 1 (anti-PD1) and anti-programmed cell death 1 ligand 1 (anti-PD-L1) antibodies have been approved by the US Food and Drug Administration (FDA) against over 15 different types of malignancies [1]. In contrast, so far, only five CAR T therapies have been approved for hematological malignancies of B cell origin (Table 1 and Fig. 1). Over 700 clinical trials of CAR T therapy have been registered at clinicaltrials.gov and many of these trials focus on solid tumors. However, no CAR therapy has been approved for solid tumors yet. This review article discusses the current status of CAR T and NK cell therapies and their limitations, followed by emerging CAR macrophage therapy.

**Table 1:** Current FDA approvals of CAR T therapies

Name	Trade name	Intra- cellular domain	Target	Approval date	Indication*	Lymphodepleting regimen	Dosing regimen**	Clinical benefit	Trial name and reference
**Axicabtagene ciloleucel**	Yescarta	CD3ζ and CD28	CD19	Oct 18, 2017	Large B-cell lymphoma	FluCy^a^	2 × 10^6^/kg	ORR: 82%, CR:54%	ZUMA-1 [2]
				Mar 5, 2021	Follicular Lymphoma	FluCy^a^	2 × 10^6^/kg	ORR: 91% CR: 60%	ZUMA-5 [3]
**Brexucabtagene autoleucel**	Tecartus	CD3ζ and CD28	CD19	Jul 24, 2020	Mantle cell lymphoma (MCL)	FluCy^a^	2 × 10^6^/kg	ORR: 93%, CR: 67%	ZUMA-2 [4]
**Idecabtagene vicleucel**	Abecma	CD3ζ and 4-1BB	BCMA	Mar 26, 2021	Multiple myeloma	FluCy^b^	150 × 10^6^	ORR: 50%, CR: 25%	KarMMa [5]
							300 × 10^6^	ORR: 69%, CR: 29%	
							450 × 10^6^	ORR: 82%, CR: 39%	
**Lisocabtagene maraleucel**	Breyanzi	CD3ζ and 4-1BB	CD19	Feb 5, 2021	Large B-cell Lymphoma	FluCy^b^	50 × 10^6^	ORR:68%, CR:60%	TRANSCEND NHL 001 [6]
							100 × 10^6^	ORR:74%, CR:52%	
							150 × 10^6^	ORR:73%, CR:51%	
**Tisagenlecleucel**	Kymriah	CD3ζ and 4-1BB	CD19	Aug 30, 2017	Acute Lymphoblastic Leukemia	FluCy^c^ OR CYVE^e^	Median: 3.1 × 10^6^/kg Range: 0.2 × 10^6^–5.4 × 10^6^/kg	Overall remission rate***: 81%. CR: 60%	ELIANA [7]
				May 1, 2018	Large B-Cell Lymphoma	FluCy^d^ OR Bendamustine (90 mg/m^2^ IV × 2 days)	Median: 3.0 × 10^8^ Range: 0.1 × 10^8^–6.0 × 10^8^	ORR: 52%; CR: 40%	JULIET [8]

*ORR* Objective response rate, *CR* Complete response, *Mo* Months, *IV* Intravenous infusion, *Kg* Kilogram of Body Weight, *FluCy* Fludarabine and Cyclophosphamide, *CYVE* Cytarabine and Etoposide

* all diseases are relapsed or refractory

** all doses are single intravenous infusions on day 0

*** defined as the rate of a best overall response of either complete remission or complete remission with incomplete hematologic recovery within 3 months

^a^ Fludarabine (30 mg/m^2^) and Cyclophosphamide (500 mg/m^2^) IV on days −5, −4, and − 3

^b^ Fludarabine (30 mg/m^2^) and Cyclophosphamide (300 mg/m^2^) IV on days − 5, − 4, and − 3

^C^ Fludarabine (30 mg/m^2^ IV × 4 days) and Cyclophosphamide (300 mg/m^2^ IV × 2 days)

^d^ Fludarabine (25 mg/m^2^) and Cyclophosphamide (250 mg/m^2^) IV on days − 5, − 4, and − 3

^e^ Cytarabine (500 mg/m2 IV × 2 days) and Etoposide (150 mg/m2 IV × 3 days)

### Main text

#### CAR structure

Optimal activation of naive T cells requires two signals, a primary signal via engagement of T cell receptor (TCR) with an antigen presented on major histocompatibility complex (MHC) and a co-stimulator signal through CD28. TCR consists of two highly variable chains, usually α and β chains for the αβ T cells or γ and δ chains in a minority of T cells. These two chains form the antigen recognition and binding site and have very short cytoplasmic tails. In addition, TCR associates with six CD3 adaptors (CD3εγ, CD3εδ and CD3ζζ) composed of four CD3 adaptor proteins, CD3δ, CD3γ, CD3ε and CD3ζ, to form an octameric complex. The CD3 adaptor intracellular domains contain immunoreceptor tyrosine-based activation motifs (ITAM) to transmit the primary signal during TCR engagement. The primary signal from the TCR-CD3 complex together with a co-signal from engagement of CD28 with B7.1 (CD80) or B7.2 (CD86) activates T cells.

**Fig. 2:**
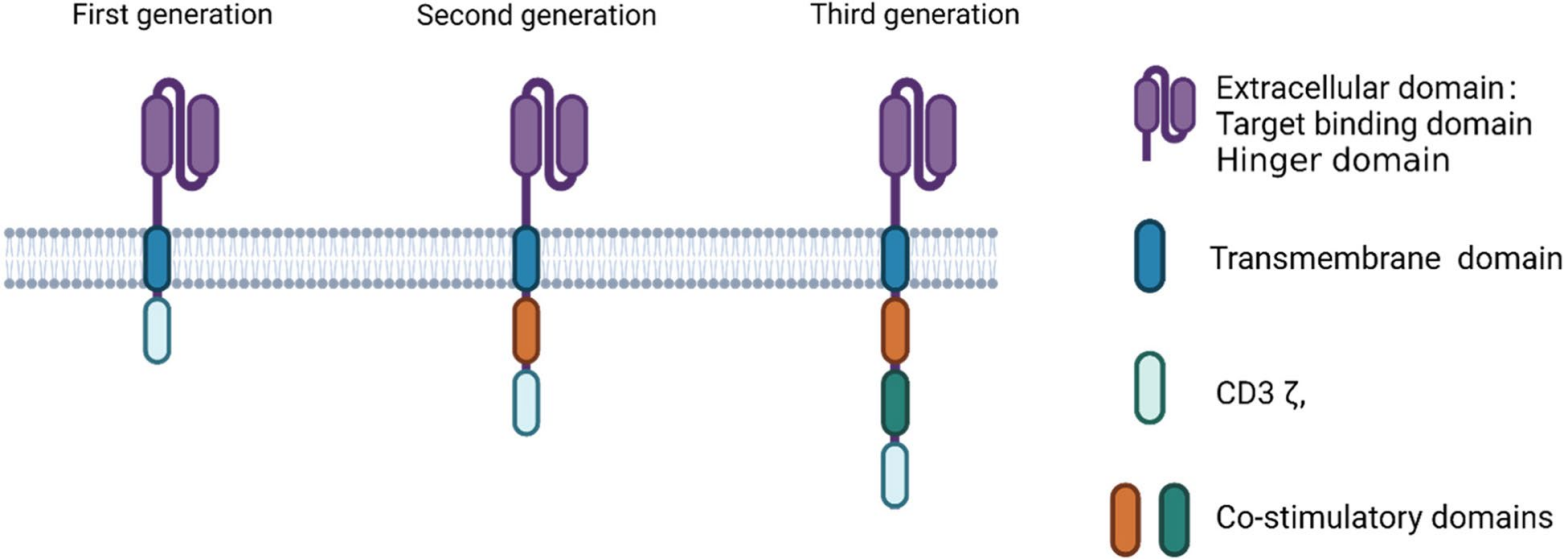
CAR structure for CAR T, CAR NK and CAR macrophage. CARs for CAR T, CAR NK and CAR macrophage have similar structures: the extracellular domain including the antigen binding domain and a spacer which is involved in engagement of target cells; transmembrane domain which docks CAR to immune cells and is also involved in other functions of CAR, such as stability and interaction with other membrane proteins; and the intracellular signaling domain which is involved in signaling transduction and activation of immune cells. For the target binding domain, in addition to scFv, native protein/peptide, cytokine and camelid nanobody have also been used. For the intracellular domain, in addition to the function to activate immune cells, other domains to regulate TME have also been used. Three generations of CAR structure are mainly determined by the difference of the intracellular domains. The first-generation CAR contains a single CD3ζ signaling domain. It has limited activities in CAR T cells as T cell activation requires a primary signal from T cell receptor complex with CD3 and a co-stimulatory signal from CD28. However, this generation of CAR has been used in CAR NK and CAR macrophage as a co-stimulatory signal is not required. The difference of the second- and third-generation CAR over the first-generation one is the addition of one and two co-stimulatory signaling domains. In the FDA-approved CAR T cells, these co-stimulatory domains are usually CD28 or 4-1BB. In CAR NK and CAR macrophage, their specific or other ITAM-containing domains are used for the intracellular signaling domain. (This figure was created at BioRender.com.)

CAR is a synthetic cell surface receptor that usually binds to a target cell surface antigen independent of MHC and redirects cytotoxic immune cells to target cells expressing that antigen. Its main function is to replace the TCR-CD3 complex and transmit the primary signal for T cell activation, as seen in the first-generation CAR, or replace both the primary signal from the TCR-CD3 complex and co-stimulatory signal from CD28 as seen in the second and third generation of CAR (Fig. 2) [9].

CAR is usually composed of four domains: extracellular antigen binding domain, spacer or hinge region, transmembrane domain and intracellular signaling domain. The antigen-binding domain is usually composed of the variable regions of antibody heavy (V_H_) and light (V_L_) chains connected via a flexible linker to form a single-chain fragment variable (scFv) and determines the binding specificity. Occasionally, instead of scFv, a native protein or peptide is used that can bind to its receptor on target cells. Unlike a TCR which recognizes an antigen presented via MHC, scFv recognizes and binds to cell surface epitope and determines the target specificity.

The hinge region is the spacer region that exposes the antigen-binding domain on CAR T cell surface for binding to target antigens. The hinge region commonly used in clinic is usually derived from CD8, CD28 or IgG. The length of the hinge region is empirically determined by the location of target antigens, with a longer hinge needed for antigens proximal to cell membrane and a shorter one for antigens exposed on cell surface.

The major function of the transmembrane domain is to dock CAR in the immune cell membrane. Some studies, however, show that this region can affect CAR expression, stability, dimerization and signal transduction [10, 11].

The intracellular signaling domain has been extensively studied in CAR engineering in order to generate CAR immune cells with the most active anti-tumor immunity. Upon engagement of CAR, it transduces signals to activate immune cells to attack target cells. The first-generation CAR T cells contain a CD3ζ signaling domain. As optimal T cell priming and activation requires both signals from TCR-CD3 and CD28 signaling pathways, it is not surprising that the first-generation CAR T cell therapy has limited efficacy and persistence after adoptive transfer [12, 13]. To improve the anticancer activity, a second-generation CAR T cell was designed which contains one co-stimulatory domain in addition to the CD3ζ signaling domain. Two of the most commonly used signaling domains are CD28 and 4-1BB (CD137) intracellular domains. The CD28 domain is used by the FDA-approved axicabtagene ciloleucel and brexucabtagene autoleucel, both targeting CD19. The 4-1BB signaling domain is used by the approved CD19-targeting lisocabtagene maraleucel and tisagenlecleucel, and by idecabtagene vicleucel which targets B cell maturation antigen (BCMA). Other intracellular co-stimulatory domains, such as OX40 [14], CD27 [15] and inducible T cell co-stimulator (ICOS) [16], have also been tested in pre-clinical studies with comparable efficacy as the CD28 and 4-1BB domains. Some of these co-stimulatory domains might have better beneficial effects on CAR T cells as one study showed CD27 co-stimulation augmented better in vivo persistence of CAR T cells than CD28 domain [17]. Their activity in CAR T cells has yet to be validated in large clinical trials.

It has been found that the intracellular CD28 and 4-1BB domains have different effects on CAR T cell differentiation and metabolism [18, 19]. A combination of 4-1BB and CD3ζ induces the central memory T cell differentiation and persistence, increases mitochondrial biogenesis, enhances fatty acid oxidation and oxidative metabolism. On the other hand, the CD28 and CD3ζ combination induces effect memory cell differentiation and glycolysis. To take advantage of different properties of co-stimulatory domains and potentiate CAR T cell efficacy, a third generation CAR T therapy is designed which juxtaposes two co-stimulatory domains in addition to CD3ζ. Combination of CD28 and 4-1BB can rescue CAR T cells with low affinity to target antigen, enhance proliferative capacity, augment central memory differentiation and improve in vivo antitumor activity [20]. When a library of CAR constructs containing variable numbers of costimulatory domains were tested, it was found that CAR T cells containing two costimulatory domains, DAP10 and CD27, achieved the best antitumor activities in vivo [21].

In addition to the basic structure of CAR, recent developments in bioengineering make it possible to further arm CAR immune cells with additional features to enhance its anticancer efficacy or to minimize its toxicity [22]. For example, chemokine receptors can be expressed on CAR T cells to facilitate chemotaxis and infiltration into tumor microenvironment; proinflammatory cytokines can be expressed to overcome immunosuppressive TME; inhibitory receptors can be knocked out to prevent CAR T cell exhaustion; dual CAR T cells can be generated to target two antigens and overcome antigen escape; and suicidal or inhibitory genes can be incorporated to prevent or minimize toxicity including on-target, off-tumor effects and cytokine release syndrome.

### Current status of CAR T cell therapies

So far, five CAR T cells have been approved by the FDA. All five target B cell surface markers, four targeting CD19 and one targeting B cell maturation antigen (BCMA) (Table 1 and Fig. 1). All five have been approved for the treatment of relapsed or refractory hematological malignancies: lymphomas and leukemias of B cell origin and multiple myeloma.

The first FDA-approved CAR T therapy is tisagenlecleucel (Kymriah™), based on a multicenter study of 75 pediatric and young adult patients with relapsed or refractory B cell precursor acute lymphoblastic leukemia (ALL) [23]. Within 3 months, it has an overall remission rate of 81% with 60% complete remission (CR) and 21% CR with incomplete hematologic recovery (CRi) [7]. Fifty five out of 75 patients (73%) had a grade 3 or 4 tisagenlecleucel-related adverse event. Grade 3 and 4 cytokine release syndrome (CRS) occurred in 21 and 25% of patients, respectively, with 35 of 75 patients (47%) being admitted to the intensive care unit (ICU) for its management. Based on this trial, CAR T therapy was approved by the FDA on August 30, 2017 for the treatment of patients ≤25 years of age with B cell precursor ALL that is refractory or in second or later relapse. Tisagenlecleucel was latter approved on May 1, 2018, for relapsed or refractory large B-cell lymphoma, including diffuse large B-cell lymphoma (DLBCL), high-grade B-cell lymphoma and DLBCL arising from follicular lymphoma, after two or more lines of systemic therapy based on a multicenter JULIET trial [8]. Of the 92 evaluable patients, the best overall response rate is 52% with 40% CR and 12% partial response (PR). Sixty three percent developed Grade 3 or 4 adverse events suspected to be related to tisagenlecleucel.

Axicabtagene ciloleucel (Yescarta™) became the second FDA approved CAR T therapy on October 18, 2017 for large B cell lymphoma, including DLBCL, primary mediastinal large B-cell lymphoma, high-grade B-cell lymphoma and DLBCL arising from follicular lymphoma, after at least two lines of systemic treatment. A multicenter ZUMA-1 trial with 101 patients showed an 82% objective response rate (54% CR) and 52% overall survival rate at 18 months [2]. Grade 3 or higher CRS occurred in about 13% of patients. On March 5, 2021, axicabtagene ciloleucel was also approved for relapsed or refractory follicular lymphoma after two or more lines of systemic therapy based on a single-arm, open-label ZUMA-5 trial. Response was achieved in 91% of patients and Grade 3 or higher CRS occurred in about 8% of patients. With a median follow-up of 14.5 months, 74% remained continued remission at 18 months [24].

The third CD-19-targeting CAR T therapy, Brexucabtagene autoleucel (Tecartus™), was approved for relapsed or refractory mantle cell lymphoma by the FDA on July 24, 2020 based on a single-arm, open-label ZUMA-2 trial [4]. In this multicenter Phase II trial with 74 patients enrolled, Brexucabtagene autoleucel was manufactured for 71 patients and administered to 68. The overall response rate was 93% with 67% CR. At 12 months, the overall survival was 83%. Grade 3 or higher CRS occurred in about 15% of patients.

Lisocabtagene maraleucel (Breyanzi™) is the most recently approved CD19-targeting CAR T therapy against relapsed or refractory large B-cell lymphoma, after two or more lines of systemic therapy, including DLBCL not otherwise specified (including DLBCL arising from indolent lymphoma), high-grade B-cell lymphoma, primary mediastinal large B-cell lymphoma, and follicular lymphoma grade 3B [6]. In this multicenter TRANSCEND trial with 192 evaluable patients, the objective response rate was 73% (68, 74 and 73% for 50 × 10^6^, 100 × 10^6^ and 150 × 10^6^ dosing regimen, respectively) with 53% CR (60, 52 and 51% for 50 × 10^6^, 100 × 10^6^ and 150 × 10^6^ dosing regimen, respectively) and the median duration of response was 17 months. Grade 3 or higher CRS occurred in about 2% of patients.

Idecabtagene vicleucel (Abecma®) is the only FDA-approved CAR T therapy not targeting CD19 [5]. It targets BCMA on multiple myeloma (MM) cells and was approved by the FDA on March 26, 2021 for relapsed or refractory MM after four or more lines of systemic therapy, including an immunomodulatory agent, a proteasome inhibitor, and an anti-CD38 monoclonal antibody based on the KarMMa study. Of 128 patients who received treatment, the overall response rate was 73% (50, 69 and 82% for 150 × 10^6^, 300 × 10^6^ and 450 × 10^6^ dosing regimen, respectively) with 33% CR (25, 29 and 39% for 150 × 10^6^, 300 × 10^6^ and 450 × 10^6^ dosing regimen, respectively). Grade 3 or higher CRS occurred in about 2% of patients.

So far, over 700 clinical trials have been registered across a wide range of malignancies. In addition, CAR T cell therapy is being explored for other diseases, such as autoimmune disease and viral infections [25].

### Limitations of CAR T cells in cancer immunotherapy

Many factors contribute to the failure of CAR T cell therapy, such as patient disease progression, insufficient harvest of T cells, delay in CAR cell manufacturing, low CAR cell production, intrinsic T cell defect and so on. The following is a list of factors associated with CAR T cells and treatment.

#### Antigen selection

Even though great success has been achieved with CAR T therapy, some of the limitations are clearly observed. So far, all the FDA-approved CAR therapies target B lineage markers. It is multifactorial why CAR T therapy in solid tumors lags behind hematological malignancies. Lack of cancer-specific targets is one of the major hurdles. Even though B cell aplasia occurs with CD19-targeting CAR T therapy, intravenous immunoglobin supplement can easily compensate most of B cell functions. There are few such specific antigens existing in solid tumors that do not affect normal functions. Clinical trials with CAR T cells targeting tumor-associated antigens in solid tumors, such as melanoma antigen recognized by T cells 1 (MART1) and glycoprotein 100 (gp100), showed that on-target off-tumor side effects do occur and sometimes cannot be easily reversed [26]. This toxicity can occur in the absence of or with minimal anti-tumor activity [27] and can lead to fatalities even when targeting cancer testes antigens which are considered not to be expressed in healthy adult tissue [28].

#### Inefficiency of CAR T cell trafficking and infiltration into tumors

CAR T cell trafficking and infiltration into tumor sites is the next major hurdle to overcome after intravenous administration. Abnormal vasculature with aberrant expression of adhesion molecules decreases CAR T cell attachment, migration and infiltration into tumor sites; dense extracellular matrix, including cancer-associated fibroblast (CAFs), creates a physical barrier for CAR T cells to enter tumor sites; and dysregulated cytokine expression preferentially attracts suppressive immune cells. Various strategies have been explored to increase T cell infiltration into tumors. CAR T therapy has been combined with another CAR T therapy targeting CAFs [29] or arming CAR T cells with a cytokine receptor binding to a cytokine upregulated in tumors [30]. However, these strategies sometimes come with a price as these targets normally exist in the host. One pre-clinical study showed that CAR T cells targeting CAFs recognize multipotent bone marrow stromal cells, which lead to cachexia and lethality in mice [31], cautioning against the use of this strategy in human patients.

#### Hostile tumor microenvironment

Upon entrance into TME, immunosuppressive milieu inhibits CAR T cell function. This is especially true in some cancers, such as pancreatic cancer [32], where both cellular and matric components create hostile microenvironment for cancer immunotherapy. Cellular components like tumor-associated macrophages (TAMs), regulatory T (T_reg_) cells, myeloid-derived suppressor cells (MDSCs) and tumor-associated fibroblasts (TAFs) can directly suppress CAR T function, contribute to immunosuppressive cytokines and metabolic microenvironment to dampen the CAR T cell function. Vascular endothelial growth factor (VEGF) plays a critical role in neovascularization of tumors. Recently it was found VEGF can affect almost every aspect of the immune system [33], leading to suppression of anticancer immunity. In addition, transforming growth factor beta (TGF-β), IL-4, IL-10 and many others also contribute to T cell dysfunction and promote infiltration of suppressive immune cells [34]. Many strategies exist and have been used to boost CAR T therapy at TME, such as combining CAR T therapy with ICIs or other immunostimulatory therapies, engineering CAR T cells to be insensitive to immunosuppressive cytokines.

#### Antigen escape

Once CAR T cells exert the anticancer activity, antigen loss and downregulation are important mechanisms of treatment failure. Despite high initial response rates, 7–25% of patients treated with CAR T therapy targeting CD19 relapse with malignancies which had diminished CD19 expression [35]. CAR T construct targeting two different antigens or sequential CAR T therapies are actively being pursued to minimize antigen escape and downregulation.

#### Insufficiency of CAR T cell expansion and persistence

In addition to target antigen escape, CAR T cell expansion and persistence in vivo are considered critical for long-term remission, especially for those malignancies that require prolonged therapy, such as ALL. It may be less critical for other malignancies, such as non-Hodgkin’s lymphoma which short intensive chemotherapy without consolidation and maintenance therapy suffices to induce remission. In the latter case, complete remission persists in spite of undetectable CAR T cells and recovery of B cells after anti-CD19 CAR T therapy [36].

Lack of CAR T cell persistence could be related to host anti-transgene immune response to CAR T cells. In this case, fludarabine-based lymphodepletion condition therapy can diminish anti-transgene immune response, improve CAR T cell expansion and persistence and enhance the efficacy of CAR T cells [37].

More commonly, lack of CAR T cell expansion and persistence are secondary to factors directly related to CAR T cells [38, 39]. Various strategies have been designed and studied to enhance CAR T cell expansion and persistence: CAR T construct, parental T cell selection, T cell culture condition, pharmacological manipulation, modification of CAR T gene expression and metabolism, reversion of T cell exhaustion, promotion of memory phenotype development and so on (reviewed at [38]). Regarding CAR T structure, most CAR T cell products being tested in clinic and approved by the FDA contain CD3ζ plus one co-stimulatory domain, usually CD28 or 4-1BB. Pre-clinical studies and some clinical observations suggest that co-stimulatory domains can affect CAR T cell phenotype and persistence in vivo with longer duration observed with 4-1BB domain, 168 days versus around 30 days with CD28-based CAR T cells [7, 18, 40–43].

Ex vivo culture, activation and expansion not only primes T cells for transduction of CAR transgenes, expands to generate sufficient CAR T cells for clinical application, but also are critical for maintaining CAR T function and persistence after infusion. Ex vivo culture that leads to T cell terminal differentiation with the predisposition toward activation-induced cell death (AICD) and exhaustion can affect in vivo expansion and persistence. Optimization of the culture condition can improve the development of memory CAR T cells and in vivo expansion and persistence [44–46].

After administration, CAR T cells may become exhausted and susceptible to AICD after repetitive antigen stimulation [47, 48]. On the other hand, contraction can occur after target malignant cells are eliminated and antigen levels are insufficient to stimulate and maintain the CAR T cell pool.

#### Systemic toxicity

CAR T cell therapy has a high response rate, especially in those resistant and refractory disease, but is also associated with a high side effect rate [49]. Systemic cytokine toxicities occur during the acute phase when CAR T cells are activated and release cytokines as a physiological reaction to CAR engagement. Severe and even lethal effects are observed with all active CAR T therapies [7, 50, 51]. For most patients, it manifests CRS associated with fever, hypotension, hypoxia and multiorgan failure associated with high levels of inflammatory cytokines. Some patients can also develop immune effector cell-associated neurotoxicity syndrome (ICANS) [52]. ICANS has distinct neurological manifestation secondary to increased cerebrospinal fluid cytokine level and disrupted blood-brain barrier, and is manifested as aphasia, altered mental status, tremor, seizure and headache [52]. Various approaches have been adopted with some already translated into clinical applications. Corticosteroids and anti-interleukin-6 (IL-6) antibody, siltuximab, are the most commonly used pharmacological intervention [53]. Several other approaches are being actively explored to control or ameliorate systemic cytokine toxicities, such as tailoring CAR T cell dose based on tumor burden, modification of CAR T construct, incorporation of off switches or suicidal genes in CAR T cells [9].

Occasionally Hemophagocytic Lymphohistiocytosis (HLH)/Macrophage Activation Syndrome (MAS) can occur after CAR T cell therapy. Based on a multi-center survey, approximately 3.5% (7/201 cases) patients treated with CAR T cell therapy develop HLH/MAS [54]. The main clinical manifestations include fever, hepatosplenomegaly, abnormal liver function, cytopenia, high ferritin level, hypertriglyceridemia and hypofibrinogenemia. Sometimes, the manifestations are similar to CRS. Several criteria have been used to diagnose HLH/MAS [54] with the HLH2004 the most commonly used at clinic [55]. Corticosteroid-based immunosuppressive therapy is the mainstay of therapy, but still with high mortality.

### Special consideration of CAR T cell therapy in solid tumors

Currently over 70% of CAR T cell clinical trials focus on hematological malignancies and over 40% on CD19 while less than 30% on solid tumors [56]. CAR T therapy in solid tumors encounters all the challenges as described above. Special considerations need to be taken when designing CAR T therapy in solid tumors.

First, target antigen selection is critical. A list of target antigens and some of the example clinical trials can be found in previous reviews and are expanding [57–59]. In CAR T therapy, the ideal target antigens are those tumor-specific antigens only expressed on malignant cells. In reality, except cancer neoantigens and possibly the epidermal growth factor receptor variant III (EGFRvIII) [60], almost all other antigens used in CAR T therapy in solid tumors are shared by normal cells. The major advantages of targeting non-cancer-specific antigens in hematological malignancies are: 1) existence of salvage remedy in case normal cells are destroyed, as seen with immunoglobin supplement with CAR T therapy targeting B cells markers (CD19 and BCMA); and 2) fast cell turnover with generation of new cells to replenish. Hence, on-target, off-tumor side effects in hematological malignancies are usually manageable. In contrast, targeting tumor-associated antigens (TAAs) in solid tumors are commonly associated with more significant side effects as it is often difficult to remedy off-tumor damage to normal cells in solid organs.

Several strategies have been designed to address lack of tumor-specific antigens in solid tumors as well as in hematological diseases. One is to split the primary and co-stimulatory signaling domains into two different CARs. Hence, these split-and CAR T cells are not robustly activated unless target cells express both target antigens [61]. A similar split-and approach is synthetic Notch (synNotch) receptor system in which binding of the first target antigen triggers the expression of CAR which can be activated by and kill cells expressing the second target in the vicinity [62]. Another approach is called the and-not approach as seen in the split, universal, and programmable (SUPRA) CAR system in which CAR can engage an TAA and activate CAR T cells, but existence of a second antigen, usually an antigen expressed on normal cells, can compete away the CAR-TAA engagement and prevent CAR T cell activation [63]. Several other approaches have also been explored, such as fine-tuning CAR affinity to achieve CAR T cell activation by cancer cells with overexpression of target antigen, but not normal cells with low antigen expression [64, 65], targeting aberrant glycosylation in cancer cells [66, 67], and using T cell receptors targeting cancer neoantigen [68].

Antigen heterogeneity is also a critical factor to consider in CAR T therapy in solid tumors. Among the FDA-approved CAR T therapies, the target antigens, CD19 and BCMA, are developmental biomarkers expressed in the stem cells that malignant cells are developed from. In solid tumors, such developmental antigens/biomarkers are shared with normal cells, and, hence, targeting these antigens can cause severe on-target, off-tumor toxicity. For non-developmental biomarkers, heterogeneous expression is commonly seen among cancer cells. To overcome target antigen heterogeneity, bi- or multi-specific CAR has been proposed in which one CAR can recognize more than one target antigen [69, 70]. Another approach is to introduce several CARs, each targeting a different antigen, into the same T cell [71]. In both approaches, on-target, off-tumor toxicity is a major concern as very few tumor-specific antigens exist.

CAR T cell infiltration into tumors and retention of cytotoxicity are major hurdles in solid tumors. These two hurdles are less issues in the case of leukemia where CAR T cells come to direct contact with target cells, or lymphoma where lymphocytes normally traffic and mount immune response in lymphoid organs. Several approaches are being explored to enhance CAR T cell activity in solid tumors [1, 72, 73]. One approach is to enhance CAR T function and arm these cells with addition molecules to augment the anti-tumor activity. For example, arming CAR T cells with a chemokine receptor enhances T cell infiltration into tumors with upregulation of the target chemokine [30, 74]. TGF-β is a major negative regulator of anticancer immunity at TME. Knockout of the TGF-β in CAR T cells enhances their anticancer activity [75]. However, a more common approach is to combine CAR T cells with another agent(s) to overcome hurdles and increase the efficacy as reviewed [72, 76]. A pharmacological-based ranking of anti-cancer drugs have been proposed that can potentially guide the development of immunotherapy combination [77].

### CAR NK cells to address unmet needs associated with CAR T therapy

NK cells are a group of cytotoxic lymphocytes of the innate immune system that can mount a rapid response to non-self cells. Unlike T cells that recognize antigens presented on MHC, NK cells can directly recognize target cells in the absence of MHC. In fact, MHC engages the killer cell immunoglobulin-like receptors (KIR) on NK cells and suppresses NK cell function. Furthermore, unlike engagement and activation of CAR T cells which can release inflammatory cytokine and lead to CRS and neurotoxicity, NK cells have different cytokine profiles. Hence, NK cells are currently being actively explored as an alternative approach for adoptive cell therapy (Table 2).

**Table 2:** Comparison of CAR T, NK and macrophages

Parameter	CAR T CELLS	CAR NK CELLS	CAR MACROPHAGES
**Intracellular signaling domain**	CD3ζ plus a costimulatory domain, CD28, 4-1BB and others	Similar to CAR T structure, but can use NK-specific signaling domains, such as 2B4, DAP10, DAP12.	Similar to CAR T structure, but can use other ITAM-containing signaling domains. Other ligands can be used not to activate phagocytosis, but to modify tumor microenvironment
**Cell source**	Autologous or MHC-matched allogeneic	Autologous, non-MHC-matched allogeneic or NK cell lines	Autologous. Preclinical studies use iPSCs and cell lines
**Off-the-shelf ready-to-use CAR product**	Unlikely. Usually autologous. Maybe MHC-matched allogeneic CAR T cells	Yes with NK cell lines. Possibly yes with allogeneic NK cells, but poor recovery with cryopreserve	Theoretically yes with macrophage cell lines. No clinical data.
**In vitro expansion**	Yes	Yes for autologous NK cells. Cell like can be pre-expanded before transducing.	Yes for autologous macrophages. iPSC and cell lines can be pre-expanded before transducing.
**Cytotoxicity mechanisms**	CAR-dependent cell killing	Both CAR-dependent and CAR-independent NK-mediated cell kiling	CAR-dependent macrophage-mediated phagocytosis; macrophage-mediated immunostimulatory TIME; macrophage-mediated alteration of tumor microenvironment; macrophages as antigen-presenting cells to stimulate immune response
**Cytokine release syndrome and neurotoxicity**	Common and often serious	Less common and serious	No clinical data. But expected to be common
**Infiltration into tumors**	Usually poor	Usually poor	Usually abundant
**Clinical experience/trial**	Proven efficacy. Five CAR T therapies approved by the FDA	Limited. No approved therapy. At least one trial has been published with superior safety profile	Very limited clinical experience. One CAR macrophage trial is ongoing.

One major advantage of CAR NK cells over CAR T cells is the source of immune cells. Due to alloreactivity and graft-versus-host disease (GVHD), CAR T therapy requires the use of autologous T cells. As these patients are usually heavily treated prior to CAR T therapy, many of them have low T cell counts at peripheral blood. Hence, harvest of sufficient autologous T cells can significantly delay the treatment, and sometimes it is not possible to harvest sufficient cells for CAR T manufacturing. In one study, 22.5% (16/71) patients had below the target autologous lymphapheresis of CD3+ T cells for the production of CAR T cells [78]. The lengthy and cumbersome process of CAR T manufacturing makes many more patients either ineligible for the treatment or have disease progression after enrolled into the treatment process. For example, in the Phase I trial of tisagenlecleucel in ALL that led to the FDA approval, of 83 patients enrolled in the trial only 54 patients received CAR T cell infusion, nine patients had progressive disease or death before treatment and 15 patients received other treatment [51]. As NK cells are not activated through the MHC pathway and have reduced risk for alloreactivity, autologous NK cells are not required for CAR NK cell manufacturing. It can use an existing NK92 cell line, umbilical cord blood and induced pluripotent stem cells (iPSCs). In fact, five clinical trials use NK-92 cell lines in human patients. In one trial using HLA-mismatched CAR NK therapy developed from cord blood, none of the 11 patients developed GVHD [79]. Hence, one major advantage of CAR NK therapy is that an “off-the-shelf” ready-to-use CAR NK cells can be manufactured through mass production and infused to patients at any time.

The second major advantage of CAR NK therapy over CAR T therapy is CRS and neurotoxicity. CAR T cell activation leads to massive release of inflammatory cytokines which cause CRS and neurotoxicity. In the Phase I trial with tisagenlecleucel in ALL, 85% (45/53) developed CRS with Grade 3 or higher toxicity occurring in 26% (14/53) of patients and 41.5% patients developed Grade 3 or higher neurotoxicity [51]. Similar toxicities are also observed with other CAR T therapies [2, 4]. Of the 11 patients treated with HLA-mismatched anti-CD19 CAR-NK cells derived from cord blood, none developed CRS or neurotoxicity [79]. The difference of these two toxicities between CAR T and NK cells may be secondary to difference in cytokines released upon cell activation. CAR T cell activation leads to release of inflammatory cytokines, such as tumor necrosis factor alpha, IL-1β, IL-2 and IL-6 among others [52], while patients receiving CAR NK therapy do not have increase of such inflammatory cytokines [79]. CAR NK cells release different profiles of cytokines, such as Granulocyte-macrophage colony-stimulating factor (*GM*-*CSF*) [80].

**Fig. 3:**
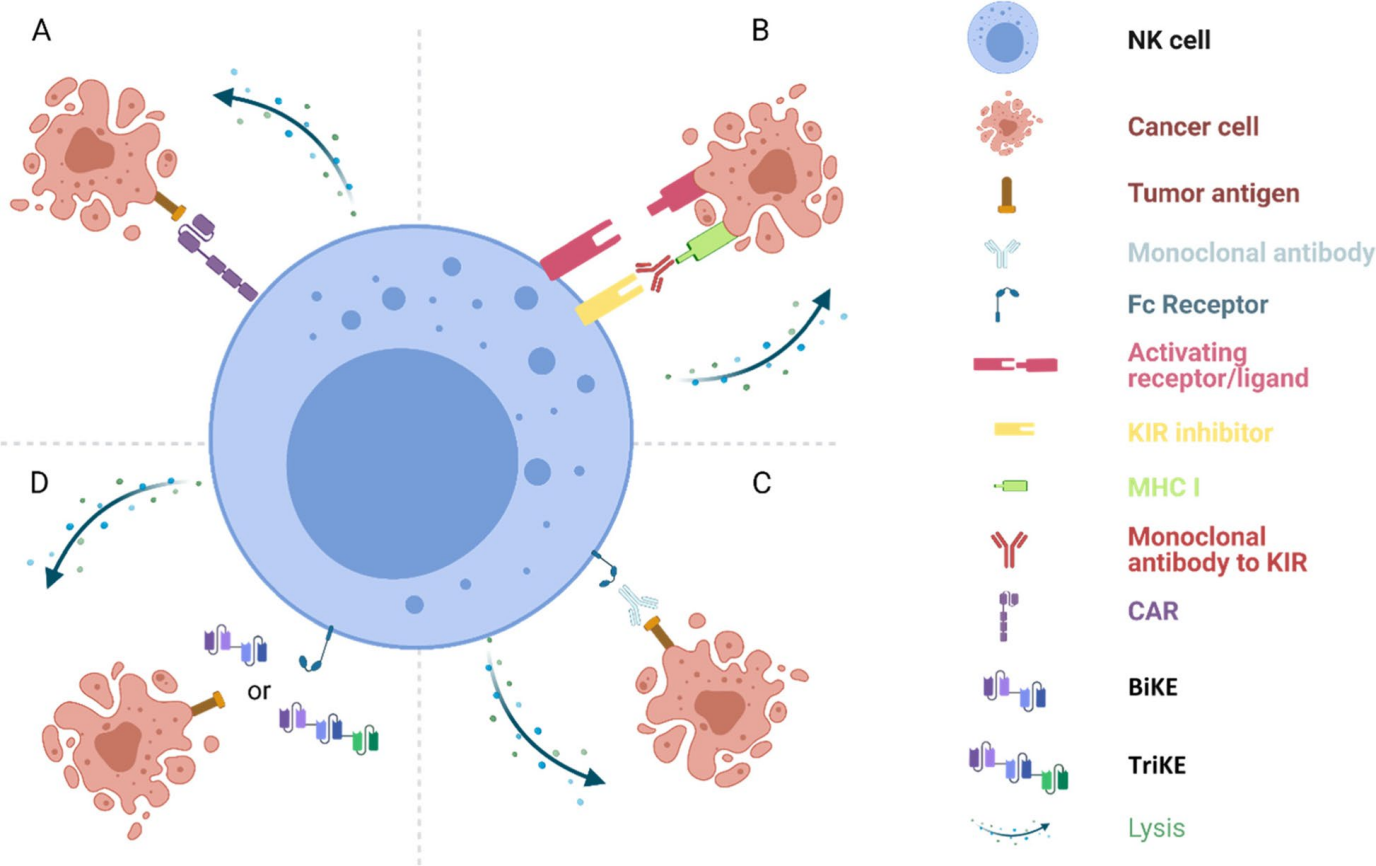
Harnessing NK cells for cancer immunotherapy. Several approaches are currently being actively pursued to exploit NK cells for cancer immunotherapy. **A** CAR NK cells. In CAR NK cells, artificial cell surface receptor on NK cells specifically recognizes tumor antigens on target/cancer cells and CAR NK cells destroy those cells. CAR can use the same CAR construct as used in CAR T cells with CD3ζ intracellular domain, or NK-specific activating domains, such as 2B4, DAP10 and DAP12. **B** Blockage of negative regulators on NK cells. The activity of NK cells is tightly regulated by both activating and inhibiting signaling pathways. The human killer cell immunoglobulin-like receptors (KIR; also known as CD158) are key negative regulators of NK cells. Engagement of KIR by MHC-I molecules on normal nucleated cells inhibits NK cell activity and induces “self” tolerance. Blockage of KIR activates NK cells to kill target cells. **C** Antibody-dependent cell-mediated cytotoxicity (ADCC). Antibody binds to its cognate antigen on target/cancer cells. Then the Fc region of the antibody is recognized by the Fc receptor, CD16, on NK cells which subsequently kills target/cancer cells coated with antibody. **D** Bi- and tri-specific killer engagers (BiKEs and TriKEs). Similar to ADCC, BiKEs and TriKEs bridge NK cells to target/cancer cells for cell killing. While the Fc portion of an antibody binds to the Fc receptor to mediate cell killing at ADCC, BiKEs and TriKEs contain a single variable portion (VH and VL) of antibody to engage the Fc receptor (CD16) on NK cells and another (for BiKE) or two other (for TriKE) variable portions of antibodies to bind to the antigen(s) on target/cancer cells. This figure was created at BioRender.com

Third, NK cells have multiple mechanisms to target and eliminate cancer cells in addition to the CAR pathway (Fig. 2). NK cells are the key mediators of antibody-dependent cell-mediated cytotoxicity through CD16 expressed on NK cells that can recognize the Fc portion of IgG bound on tumor cells and kill cancer cells. Furthermore, NK cells can be activated to kill cancer cells through engagement and/or disengagement of the activating and inhibitory killer Ig-like receptors (KIRs) on cell surface. The activating and inhibitory KIRs transduce their signals through ITAMs (immunoreceptor tyrosine-based activation motif) and ITIMs (immunoreceptor tyrosine-based inhibition motif), respectively. Cells in stress and cancer cells downregulate MHC class I expression and disengage the inhibitory KIRs, or upregulate stress-induced molecules, such MICA/MICB (MHC class I chain-related protein A/B), to engage activating KIRs that tilts the activation of NK cells to kill target cells [81, 82] Fig. 3.

Fourth, NK cells have limited lifespan. The average lifespan of NK cells is approximately 2 weeks [83]. This means that, in case of on-target off-tumor toxicity occurs, it can self-limit with the disappearance of CAR NK cells. This however, also creates a double-edge sword that repeated infusion of CAR NK cells may be needed for prolonged remission.

### Comparison of CAR constructs

So far, most CARs used for CAR NK cell studies use the same CAR constructs as used in CAR T cells with the same intracellular domains of CD3ζ as in the first-generation CAR T cells [84], or CD3ζ plus a co-stimulatory domain 4-1BB as used in the second-generation CAR T cells [85] (Fig. 1). Addition of a 4-1BB co-stimulatory domain significantly improves NK cell activation, cytotoxicity and production of cytokines, such as interferon-γ and granulocyte-macrophage colony-stimulating factor.

As NK cells have their own intertwined activating and inhibitory receptors to control NK cell activation and cytotoxicity, it is proposed that using NK-specific intracellular signaling domains, such as the adapter molecule DAP10 or ITAM-containing signaling domains, such as DAP12 and 2B4, may improve cytotoxicity. 2B4 belongs to the signaling lymphocytic activation molecule (SLAM) family receptor and transduces activation signal through SLAM-associated protein (SAP) in NK cells [86]. Engagement of 2B4 with CD48 on target cells induces NK cell activation with increased cytotoxicity and interferon-gamma production. Compared to CAR NK cells containing 4-1BB-CD3ζ, CAR NK cells containing 2B4- CD3ζ have improved cytotoxicity, interferon gamma production and in vivo anti-tumor efficacy [87].

To further optimize the construct of CAR for NK cells, Li et al. compared one CAR T cell construct with nine different CAR NK cells containing four different transmembrane domains and different intracellular signaling domains targeting the same mesothelin antigen [88]. CAR NK cells containing the NKG2D transmembrane domain, 2B4 co-stimulatory domain and CD3ζ signaling domain exhibit strong antigen-specific cytotoxicity. CAR NK cells developed from human iPSC carrying this construct have a typical NK cell phenotype, exert significant anti-tumor activity and have prolonged survival in vivo.

### Current status of CAR NK cell therapy

So far, at least 24 clinical trials with CAR NK cells have been planned or are ongoing. The malignancies, target antigens and clinical trial information are shown at Table 3 and Supplement Information 1. All these clinical trials are at the Phase I/II trial stage.

**Table 3:** Clinical trials with CAR NK cells

#	Clinical Trial identifier	Status	Clinical trial phase	Disease	Antigen	Interventions	Dosage	starting time
1	NCT03692663	Not yet recruiting	Early Phase 1	Castration-resistant Prostate Cancer	PSMA	anti-PSMA CAR NK cells	0.5-3 × 10^6/kg	Dec-18
2	NCT03690310	Not yet recruiting	Early Phase 1	Refractory B-Cell Lymphoma	CD19	Anti-CD19 CAR NK Cells	50-600 × 10^3/kg	Mar-19
3	NCT03692767	Not yet recruiting	Early Phase 1	Refractory B-Cell Lymphoma	CD22	Anti-CD22 CAR NK Cells	50-600 × 10^3/kg	Mar-19
4	NCT03692637	Not yet recruiting	Early Phase 1	Epithelial Ovarian Cancer	Mesothelin	anti-Mesothelin Car NK Cells	0.5-3 × 10^6/kg	Mar-19
5	NCT04639739	Not yet recruiting	Early Phase 1	NHL	CD19	anti-CD19 CAR NK	2 × 10^6/kg, 6 × 10^6/kg, 2	Dec-20
6	NCT04847466	Not yet recruiting	Phase 2	Gastroesophageal Junction (GEJ) Cancers|Advanced HNSCC	PD-L1	PD-L1 t-haNK	2 × 10^9	Jul-21
7	NCT03056339	Recruiting	Phase 1/2	B-Lymphoid Malignancies|Acute Lymphocytic Leukemia|Chronic	CD19	iC9/CAR.19/IL15-Transduced CB-	1 × 10^5	Jun-17
8	NCT03383978	Recruiting	Phase 1	Glioblastoma	HER2	NK-92/5.28.z	1 × 10^7-1 × 10^8	Dec-17
9	NCT04887012	Recruiting	Early Phase 1	B-cell Non Hodgkin Lymphoma	CD19	anti-CD19 CAR-NK	unknown	Mar-19
10	NCT03940833	Recruiting	Phase 1/2	Multiple Myeloma	BCMA	BCMA CAR-NK 92 cells	unknown	May-19
11	NCT03940820	Recruiting	Phase 1/2	Solid Tumor	ROBO1	ROBO1 CAR-NK cells	unknown	May-19
12	NCT03941457	Recruiting	Phase 1/2	Pancreatic Cancer	ROBO1	BiCAR-NK cells (ROBO1 CAR-NK ce	unknown	May-19
13	NCT03931720	Recruiting	Phase 1/2	Malignant Tumor	ROBO1	BiCAR-NK/T cells (ROBO1 CAR-NK	unknown	May-19
14	NCT04245722	Recruiting	Phase 1	Lymphoma, B-Cell|Chronic Lymphocytic Leukemia	CD19	Drug: FT596|Drug: Cyclophospha	unknown	Mar-20
15	NCT04555811	Recruiting	Phase 1	NHL|Non Hodgkin Lymphoma|Diffuse Large B Cell Lymphoma|Hig	CD19	Drug: FT596|Drug: Rituximab	(Dose Level 1: 9 × 10^7 c	Sep-20
16	NCT04623944	Recruiting	Phase 1	Relapsed/Refractory AML|AML, Adult|MDS|Refractory Myelodys	NKG2DL	NKX101 - CAR NK cell therapy	Part 1/Regimen A: 1 × 10	Sep-20
17	NCT04747093	Recruiting	Phase 1/2	B Cell Leukemia|B Cell Lymphoma|B-cell Acute Lymphoblastic Leu	unknown	CaR-ITNK cells	unknown	Jan-21
18	NCT04796675	Recruiting	Phase 1	Acute Lymphocytic Leukemia|Chronic Lymphocytic Leukemia|No	CD19	CAR-NK-CD19 Cells	0.01 × 10^7, 0.1 × 10^7, 1.	Apr-21
19	NCT02742727	Unknown status	Phase 1/2	Acute Myeloid Leukemia|Precursor T-Cell Lymphoblastic Leukemi	CD7	anti-CD7 CAR-pNK cells	unknown	Mar-16
20	NCT02839954	Unknown status	Phase 1/2	Hepatocellular Carcinoma|Non-small Cell Lung Cancer|Pancreatic	MUC1	anti-MUC1 CAR-pNK cells	unknown	Jul-16
21	NCT02892695	Unknown status	Phase 1/2	Acute Lymphocytic Leukemia|Chronic Lymphocytic Leukemia|Fol	CD19	anti-CD19 CAR-NK cells	unknown	Sep-16
22	NCT02944162	Unknown status	Phase 1/2	Acute Myelogenous Leukemia|Acute Myeloid Leukemia|Acute	MCD33	anti-CD33 CAR-NK cells	unknown	Oct-16
23	NCT03415100	Unknown status	Phase 1	Solid Tumours	NKG2DL	CAR-NK cells targeting NKG2D liga	unknown	Jan-18
24	NCT03824964	Unknown status	Early Phase 1	Refractory B-Cell Lymphoma	CD19/CD22	Anti-CD19/CD22 CAR NK Cells	50-600 × 10^3/kg	Feb-19

Using “chimeric” and “NK” the key words, only one clinical trial has been identified at PubMed (Table 4). Liu et al. published a Phase I clinical trial with CAR NK cells targeting CD19-positive lymphoid tumors [79]. In this trial, a similar CAR structure as CAR T cells was used to establish CAR NK cells with anti-CD19 scFv as the antigen-binding extracellular domain and CD3ζ as the intracellular signaling domain. In addition, CAR NK cells express interleukin-15 to enhance the in vivo expansion of CAR NK cells, and rimiducid-inducible caspase-9 as a safety measure to trigger apoptosis of the CAR NK cells in the event of severe adverse events [89, 90]. Eleven patients with B-lymphoid malignancies were enrolled into the trial. These patients received a median of four lines of therapy prior to enrollment. CAR NK cells were generated from cord blood specimens with partially matched HLAs (the first 9 patients) or without consideration of HLA matching. None of the 11 patients developed CRS, neurotoxicity, hemophagocytic lymphohistiocytosis or graft-versus-host disease. No increase of inflammatory cytokines was observed in any of these patients. Eight out of 11 patients (73%) had an objective response, including 7 CRs (three with CLL and 4 with lymphoma) and one complete remission of high-grade lymphoma in a patient with Richter’s transformation from CLL. CAR NK cells could persist for at least 12 months.

**Table 4:** Summary of a Phase I CAR NK cell clinical trial targeting CD19

NK source	Target antigen	CAR structure				Additional structure	Vector	Total patients	Diseases	Major findings
		CAR structure	Spacer	TM domain	Intracellular domain					
HLA-unmatched cord blood	CD19	Anti-CD19 scFV	IgG1-CH2CH3 portion	CD28	CD3ζ	IL-15, inducible caspase 9	retroviral	11	CLL: 4; CLL with Richter’s transformation: 1; follicular lymphoma: 4; DLBCL: 2	• No GVHD • No cytokine releasing syndrome, neurotoxicity, hemophagocytic lymphohistiocytosis • 8/11 complete response • CAR NK cells persisted ≥12 months

### Limitations associated with CAR NK cells

While the advantages of CAR NK cell therapy over CAR T cell therapy are obvious as discussed above, significant limitations also exist. Almost all the limitations associated with CAR T therapy also apply to CAR NK cells, from target antigen selection, antigen heterogeneity, CAR design, manufacturing to post-infusion challenges, such as NK cell migration into tumor sites, hostile tumor microenvironment. Active research is currently ongoing to improve CAR NK manufacturing and storage, especially for “off-the-shelf” CAR NK cells, optimize CAR NK structure to increase CAR NK infiltration into tumors and maintain cytotoxicity at the immunosuppressive TME, and minimize on-target, off-tumor toxicity. Furthermore, NK cell, have a short half-life (< 10 days) [83] which is a double-edge sword during CAR NK therapy. This confers an advantage in case severe toxicity occurs, but also creates a challenge that repeated administrations may be needed to achieve durable response. Reprogramming CAR NK cells with memory cell property and long-term survival in vivo for continuous immune surveillance and prevention of cancer recurrence is an area of active exploration now. In addition, similar to T cells, NK cells have both activating and inhibitory KIRs on cell surface. The universally expressed MHC molecules on nucleated cells can inhibit NK cell function.

### CAR macrophage to address unmet needs associated with CAR T and CAR NK therapy

High success of CAR T therapy has already been achieved in hematological malignancies, but it has yet to come to fruition in solid tumors even with strong pre-clinical studies. Some obvious differences exist between hematological and solid malignancies. For example, rare target antigens exist in solid tumors as observed with CD19 in B cell malignancies. The dramatic difference may also be secondary to the underlying physiological differences between hematological and solid tumors. When the cancer immunity cycle is considered [91], CAR T cells bypass the first three steps of the cycle: cancer antigen release and presentation, T cell priming and activation. However, the efficacy of CAR T cells is still governed by the last four steps of anticancer immunity, i.e. T cell trafficking (Step 4) and infiltration (Step 5) into tumors, recognition (Step 6) and killing of cancer (Step 7). As discussed above, these four steps create tremendous hurdles for T cells. For example, many solid tumors are immune “cold” tumors which have very little immune cell infiltration into TME. Due to immunosuppressive TME, even in those cancers that have significant immune cell infiltration, immune cells are not able to eradicate cancer cells.

Even though there are several advantages associated with CAR NK cells over CAR T cells, most hurdles associated with CAR T cells are also applicable to CAR NK cells. NK cells are usually not a major immune cell population existing in TME. Arming CAR NK cells can address some of the hurdles. For example, arming CAR NK cells with IL-15 can significantly enhance the in vivo expansion and persistence of CAR NK cells, improve the anti-tumor activity and overall survival in mouse models [90]. Since there is only one Phase I clinical trial reported so far [79], it is not clear whether arming CAR NK cells with cytokines can be generalized to other malignancies. Once NK cells enter the tumor sites, the immunoinhibitory TME can suppress NK cell functions.

Due to the hurdles associated with CAR T and CAR NK cell therapies, recently CAR macrophages have emerged as an alternative therapy. CAR macrophages share many features and hurdles as CAR T cells, such as requirement of specific antigen, antigen escape and downregulation, and systemic cytokine toxicities. However, CAR macrophages possess unique advantages over CAR T cells on two other major hurdles in solid tumors: immune cell trafficking and infiltration into TME, and immunosuppressive TME (Table 2).

For immune cell trafficking and infiltration into TME, in contrast to poor infiltration of T cells, macrophages exist abundantly in many tumors. Fresh frozen tumor sections revealed that macrophages account for most of tumor-infiltrating cells in many cancer types, up to 50% as seen in melanoma, renal cell cancer and colorectal cancers [92]. Infiltration of macrophages at TME is secondary to many cytokines secreted at the tumor sites. Hypoxia induces tumor cells and stroma to produce cytokines, such as CCL2 (C–C motif chemokine ligand 2), CXCL12 (C-X-C Motif Chemokine Ligand 12), CSF1 (Colony Stimulating Factor 1) and VEGF to recruit macrophages. Once recruited into the hypoxic TME, the receptors for these soluble factors are downregulated which locks macrophages at TME [93]. Furthermore, macrophages can directly sense hypoxic condition and its metabolites, such as low pH, and migrate into TME.

Another feature at TME that posts a major hurdle for T cells, but less so for macrophage is immunosuppressive microenvironment. Because of this environment, T cells that infiltrate into tumor sites often develop exhaustion phenotypes and sometimes cannot be reversed with immune checkpoint inhibitors. This may not be the case for macrophage. Macrophages are broadly classified into two major groups, the classically activated proinflammatory M1 macrophages and alternatively activated anti-inflammatory M2 macrophages [94]. TAMs, especially M2 TAMs, are widely considered as one of the central immunosuppressive cell populations at TME [95]. Even though M2 macrophages suppress the function of other immune cells, they still possess the phagocytosis capacity. In fact, M2 macrophages have higher phagocytic capacity than M1 macrophages [96]. Furthermore, macrophages possess a higher degree of phenotype plasticity which allows them to respond to environmental stimuli and change the phenotype.

### CAR structure in CAR macrophages

CAR in CAR macrophages has the same structure as that in CAR T cells with an extracellular antigen-binding domain, hinge region, transmembrane domain and intracellular domain (Fig. 1). They differ in the intracellular signaling domain. CAR macrophages can directly use the CD3ζ intracellular domain as used in CAR T cells which contains immunoreceptor tyrosine-based activation motifs (ITAMs) [97–99]. In CAR T cells, ITAMs are phosphorylated by Src family kinases upon CAR engagement, bind to tandem SH2 (tSH2) domains in the kinase ZAP70, and activate CAR T cells to exert cytocidal effects. Macrophages do not express ZAP70. They express another kinase Syk which contains tSH2 domains, can bind to CD3ζ and transduce phagocytic signals in macrophages [100].

In addition to CD3ζ, other ITAM-containing intracellular domains, such as the γ subunit of Fc receptor (FcRγ) and multiple epidermal growth factor-like domains protein 10 (Megf10), have also been used and can induce comparable phagocytosis as CD3ζ [97, 98]. FcRγ transduces canonical signaling for antibody-dependent cellular phagocytosis (ADCP) in macrophages. Megf10 plays an critical role in phagocytosis of apoptotic cells by macrophages [101].

Similar to the second- and third-generation CAR T cells, an additional signaling domain enhances phagocytosis. CAR T cells containing the CD3 domain without a co-stimulatory domain, as seen in the first generation CAR T cells, have limited in vivo activity [102]. Hence, all the FDA-approved CAR T products contain a co-stimulatory intracellular domain, either CD28 or 4–1 BB (Table 1). Similar findings have been observed in CAR macrophages. It was previously reported that phosphoinositide 3-kinase (PI3K) signaling is important for phagocytosis of large particles [103]. A tandem fusion of the CD19 PI3K-recruiting domain to CAR FcRγ tripled the phagocytosis of target whole cells [97].

### Current status of CAR macrophage development-preclinical studies

So far, CAR macrophage research is mainly at the pre-clinical stage with one Phase I trial ongoing which uses autologous CAR macrophages targeting HER2 overexpressing solid tumors (Table 5).

**Table 5:** Current status of CAR macrophage studies

References	Macrophage source	Target antigens	Extracellular/ Intracellular domains	Major findings
**Morrisey et al** [97]	J774A.1 Macrophages	CD19, CD22	Extra: scFv Intra: Megf10, FcγR, CD3ζ, FcγR + PI3K	1. ITAM-containing CD3ζ, FcRγ and Megf10 intracellular domains have comparable phagocytic efficiency 2. Addition of a PI3K-recruiting domain enhances phagocytosis of whole cells 3. Most CAR macrophages exert trogocytosis 4. Addition of anti-CD47 antibody enhances phagocytosis
**Zhang L. et al** [104]	induced pluripotent stem cells (iPSCs)	CD19	Extra: scFv Intra: CD86 + FcγRI	1. iPSCs can be used to generate CAR macrophages 2. iPSCs CAR macrophages (CAR-iMACs) possess M2 phenotype 3. Engagement of target cells titls toward M1 differentiation 4. CAR-iMACs can expand, persist and exert anti-tumor activities *in vivo*
**Zhang W. et al.** [105]	Raw264.7 monocyte/ macrophages	HER2	Extra: scFv Intra: CD147	1. CAR-147 upregulates MMP expression *in vitro* and *in vivo*; 2. CAR-147 does not exhibit phagocytosis; 3. CAR-147 decreases collagen content, induces CD3+ T cell infiltration and inhibits tumor growth.
**Niu et al.** **[**99**]**	Raw264.7 monocyte/ macrophages	CCR7	Extra: CCL19 Intra: TLR2, TLR4, TLR6, MerTK, 4-1BB-CD3ζ	1. CAR-M (MerTK) has the most efficient cell killing and phagocytosis *in vitro* compared to CAR-M with other intracellular domains; 2. At high dose, CAR-M also induces hair and body weight loss as CCR7 is also expressed at hair follicles and intestinal villi; 3. CAR-M (MerTK) suppresses tumor growth, prolongs survival, inhibits cancer metastasis in mice with little toxicity; 4. CAR-M (MerTK) significantly induces CD3+ T cell infiltration, decreases PD-L1-positive cells, and increases pro-inflammatory cytokine production in tumors.
**Klichinsky et al** **[**98**]**	Human THP-1 cell line	HER2	Extra: scFv Intra: CD3ζ	1. A replication-incompetent adenoviral vector can highly efficiently deliver CAR to human macrophages; 2. Adenoviral injection induces M1 differentiation and pro-inflammatory tumor microenvironment; 3. Adenovirus-transduced CAR macrophages can cross-present tumor-derived antigens and more efficiently activate T cells 4. Adenovirus-transduced CAR macrophages significantly prolongs survival and decreases metastasis of tumor-carrying mice.

Morrissey et al. systemically analyzed intracellular domains that can be used to construct CD19- and CD22-targeting CAR macrophages [97]. CAR macrophages with any of the ITAM-containing intracellular domains, CD3ζ, FcRγ or Megf10, had comparable phagocytic efficiency while CAR macrophages containing Bai1 and MerTK intracellular domains could not bind to target beads. Most CAR macrophages only internalized fragments of target cells, a phenomenon resembling trogocytosis or nibbling of live cells. Whole cell engulfment was infrequent. An antibody blocking the “don’t eat me” signal CD47 or CAR containing FcRγ-PI3K-recruiting domains enhanced phagocytosis of whole cells, but still less than 10% of macrophages contained whole cells after 4–8 h of incubation.

Klichinsky et al. reported generation and characterization of CD3ζ-based anti-HER2 CAR macrophages [98]. In this study, a replication-incompetent adenoviral vector was used to deliver CAR to macrophages with high efficiency and reproducibility. Adenoviral infection induced M1 differentiation of CAR macrophages as well as tilted TME toward a proinflammatory state instead of an anti-inflammatory M2 state associated with most tumors. Furthermore, adenovirus-transduced CAR macrophages, as professional antigen-presenting cells, could cross-present tumor-derived antigens in addition to target antigens and more efficiently activate T cells. Consistent with these in vitro findings, these CAR macrophages significantly prolonged survival and decreased lung metastasis of mice carrying tumor implants.

A study reported by L. Zhang et al. addresses the issue of inefficiency in bioengineering macrophages for cancer immunotherapy by using induced pluripotent stem cells (iPSCs)-derived CAR macrophages [104]. In this study, non-integrating episomal vectors encoding reprograming factors were used to induce iPSC clones. Then CAR containing CD86 and FcRγ intracellular domains was transduced into iPSC-induced macrophages. These iPSC-derived CAR-expressing macrophages (CAR-iMACs) possess M2 phenotype. However, upon encounter with target cells, these CAR-iMACs could engulf the target cancer cells and were tilted toward a pro-inflammatory M1 state. In vivo studies showed that CAR-iMACs could expand, persist and exert their anti-tumor activities.

W. Zhang et al. used CAR macrophages to address the insufficient immune cell infiltration into tumors caused by extracellular matrix (ECM) [105]. ECM creates a physical barrier for T cells to infiltrate into tumor sites and exert their anticancer immunity. CAR T cells targeting cancer-associated fibroblasts or matrix-degrading enzyme, heparinase, can promote immune cell infiltration and augment anticancer immunity [29, 106]. In Zhang’s CAR macrophages, scFv targeting HER2 is conjugated to a hinge region and CD147 transmembrane and intracellular domains to generate CAR-147 macrophages. CD147 is essential for ECM remodeling via the expression of matrix metalloproteinases (MMPs) [107]. Since CD147 does not transduce phagocytic signal, engagement of CAR macrophages with target cells significantly upregulated the expression of certain MMPs, but did not affect other macrophagic functions, such as phagocytosis, reactive oxygen species (ROS) production and inflammatory cytokine secretion. Consistent with the findings in vitro, CAR-147 macrophages upregulated the expression of a few MMPs, significantly reduced collagen content in tumors, induced CD3+ T cell infiltration and inhibited tumor growth.

Niu et al. reported a study of using CAR macrophage to target CCR7-expressing immunosuppressive cells for cancer immunotherapy [99]. This group previously found that lipid droplet high (LD^hi^) immunosuppressive cells expressed CC-chemokine receptor 7 (CCR7), accumulated in tumor tissues, and suppressed anticancer immunity [108, 109]. Hence, CAR macrophages were designed that express the CCR7 nature ligand CCL19 (C-C Motif Chemokine Ligand 19) as the extracellular domain to target CCR7-expressing immunosuppressive cells. To develop an optimal CAR construct, the following intracellular domains were used and compared: TLR2, TLR4, TLR6, MerTK, or the classical CAR-T activation domain, 4-1BB-CD3ζ. CAR macrophage containing MerTK or CAR-M (MerTK) had the strongest phagocytosis and cell killing activities in vitro. Since CCR7 is also expressed at hair follicles and intestinal villi, on-target off-tumor toxicities were observed when high-dose CAR-M (MerTK) was administered. At low doses, it induced CD3+ T cell infiltration into tumors, increased pro-inflammatory cytokine production, suppressed tumor growth, prolonged overall survival and decreased metastasis with little toxicity in syngeneic BALB/c mice carrying 4 T1 tumors.

### Current status of CAR macrophage development-clinical development

So far, there is only one Phase I clinical trial with CAR macrophages (Clinicaltrials.gov identifier number: NCT04660929). This clinical trial is based on the CAR macrophages developed by Klichinsky et al. as discussed above [98]. This trial uses CAR macrophages engineered with chimeric adenoviral vector Ad5f35 and carrying scFv targeting HER2. Adenoviral infection induces the differentiation of macrophages into a pro-inflammatory M1-like phenotype. This clinical trial started in February 2021. So far, no results have been reported yet. A second study with CAR macrophage was also registered (Clinicaltrials.gov identifier number: NCT05007379). This is not a clinical trial. This study is an observation study to determine the anti-tumor activity of CAR macrophages in patient-derived organoids from 100 patients.

### Limitations associated with CAR macrophages

CAR macrophage is still at its nascent stage with only one clinical trial initiated and no results reported yet. Hence, many of the limitations have yet to be unfolded. Similar to CAR T and NK cells, CAR macrophages will need to go through 7 steps along the cancer-immunity cycle to achieve the cytotoxicity effects. Great endeavors are under way to optimize CAR macrophage structure, manufacturing, storage, tumor infiltration, and retention to cytotoxicity at TME. Repeated dosing may be needed to maintain sufficient CAR macrophage levels for active cancer surveillance. One major advantage of using macrophages for ACT is its propensity in migration and infiltration into tumors [92]. With the plasticity of inter-differentiation between pro-inflammatory M1 and anti-immune M2 phenotypes, high infiltration of macrophages into tumors and differentiation into the M2 phenotype can promote cancer growth and metastasis. Differentiation and retention of the M1 phenotype is being explored.

## Conclusions

In summary, ACT with CAR T therapy has made tremendous progress in hematological malignancies with five CAR T therapies approved by the FDA so far. CAR T therapy in solid tumors lags behind secondary to lack of cancer-specific antigen, low efficiency of CAR T cell trafficking and migration into tumor sites, immunosuppressive TME among others. CAR NK cells have also been studied and translated into clinical trials [110]. There are several advantages of CAR NK cells. A limited lifespan of NK cells means lower risk of on-target/off-tumor toxicity; different cytokine profile released by NK cells represents a diminished risk for cytokine release syndrome and neurotoxicity; and reduced risk for alloreactivity allows generation of off-the-shelf allo-CAR NK cells using NK cell lines. Macrophages are among the major infiltrated cells at TME. Even immunosuppressive M2 macrophages possess strong phagocytic activity. Recently CAR macrophages are being explored as an alternative approach for the ACT. Preclinical studies have shown promising anti-tumor activity with one clinical trial with CAR macrophages targeting HER2-expressing solid tumors ongoing. Future CAR macrophage therapy still needs to overcome some other obstacles encountered with CAR T therapy. Since tumor-associated macrophages are the major cell type and main immune regulator at TME, one major research direction is to develop CAR macrophage not only as a phagocytic machinery but more importantly, an antigen presenter, TME modifier and immune stimulator to promote anticancer immunity.

## Supplementary Information

**Additional file 1.**

## Data Availability

Data sharing is not applicable to this article as no datasets were generated or analyzed during the current study.

## Abbreviations

ACT: adoptive cell therapy
ADCP: antibody-dependent cellular phagocytosis
ALL: acute lymphoblastic leukemia
BCMA: B cell maturation antigen
CAF: cancer-associated fibroblast
CAR: chimeric antigen receptor
CAR-iMAC: iPSC-derived CAR-expressing macrophage
CAR-M (MerTK): CAR macrophage containing MerTK
CCL19: C-C Motif Chemokine Ligand 19
CCL2: C–C motif chemokine ligand 2
CCR7: CC-chemokine receptor 7
CR: complete remission
CRi: complete remission with incomplete hematologic recovery
CRS: cytokine release syndrome
CSF1: Colony Stimulating Factor 1
CTLA4: Cytotoxic T Lymphocyte-Associated Protein 4
CXCL12: C-X-C Motif Chemokine Ligand 12
DAP10: DNAX-activation protein 10
DLBCL: diffuse large B-cell lymphoma
ECM: extracellular matrix
FcRγ: the γ subunit of Fc receptor
FDA: the Food and Drug Administration
HER2: human epidermal growth factor receptor 2
ICANS: immune effector cell- associated neurotoxicity syndrome
ICI: immune checkpoint inhibitor
ICOS: inducible T cell co-stimulator
IL-6: interleukin-6
iPSC: induced pluripotent stem cell
ITAM: immunoreceptor tyrosine-based activation motif
LD^hi^: lipid droplet high
MDSC: myeloid-derived suppressor cell
MHC: major histocompatibility complex
MMP: matrix metalloproteinase
Mo: Months
NK cell: nature killer cells
NKG2D: the nkg2 family of C-type lectin-like receptor D
ORR: Objective response rate
PD1: anti-programmed cell death 1
PD-L1: programmed cell death 1 ligand 1
Pi3k: phosphatidyl inositol 3 kinase
ROS: reactive oxygen species
scFv: single-chain fragment variable
TAA: tumor-associated antigens
TAF: tumor-associated fibroblasts
TAM: tumor-associated macrophage
TCR: T cell receptor
TGFβ: transforming growth factor beta
TME: tumor microenvironment
VEGF: Vascular endothelial growth factor
